# Persistently high proportions of *plasmodium*-infected *Anopheles funestus* mosquitoes in two villages in the Kilombero valley, South-Eastern Tanzania

**DOI:** 10.1016/j.parepi.2022.e00264

**Published:** 2022-08-03

**Authors:** Salum A. Mapua, Emmanuel E. Hape, Japhet Kihonda, Hamis Bwanary, Khamis Kifungo, Masoud Kilalangongono, Emmanuel W. Kaindoa, Halfan S. Ngowo, Fredros O. Okumu

**Affiliations:** a Environmental Health and Ecological Sciences Department, Ifakara Health Institute, P. O. Box 53, Morogoro, Tanzania; b School of Public Health, Faculty of Health Sciences, University of the Witwatersrand, Johannesburg, South Africa; c Centre for Applied Entomology and Parasitology, School of Life Sciences, Keele University, Huxley Building, Keele, Staffordshire ST5 5BG, UK; d Institute of Biodiversity, Animal Health and Comparative Medicine, University of Glasgow, Glasgow G12 8QQ, UK; e School of Life Science and Bioengineering, The Nelson Mandela African Institution of Science and Technology, P. O. Box 447, Arusha, Tanzania

**Keywords:** *Anopheles funestus*, Kilombero valley, Malaria transmission, *Plasmodium*, Sporozoite prevalence, Ifakara health institute, Tanzania, CDC, Centers for Diseases Control and Prevention, LLINs, Long-Lasting Insecticidal Nets, NMSP, National Malaria Strategic Plan, ITN, Insecticide-Treated Net, IRS, Indoor Residual Spraying, ELISA, Enzyme-Linked Immunosorbent Assay, GLM, Generalized Linear Model, GLMM, Generalized Linear Mixed Model, *Pf*-CSP, *Plasmodium falciparum* Circumsporozoite Protein, PCR, Polymerase Chain Reaction

## Abstract

**Background:**

In south-eastern Tanzania where insecticide-treated nets have been widely used for >20 years, malaria transmission has greatly reduced but remains highly heterogenous over small distances. This study investigated the seasonal prevalence of *Plasmodium* sporozoite infections in the two main malaria vector species, *Anopheles funestus* and *Anopheles arabiensis* for 34 months, starting January 2018 to November 2020.

**Methods:**

Adult mosquitoes were collected using CDC-light traps and Prokopack aspirators inside local houses in Igumbiro and Sululu villages, where earlier surveys had found very high densities of *An. funestus*. Collected females were sorted by taxa, and the samples examined using ELISA assays for detecting *Plasmodium* circumsporozoite protein in their salivary glands.

**Results:**

Of 7859 *An. funestus* tested, 4.6% (*n* = 365) were positive for *Pf* sporozoites in the salivary glands. On the contrary, only 0.4% (*n* = 9) of the 2382 *An. arabiensis* tested were positive. The sporozoite prevalence did not vary significantly between the villages or seasons. Similarly, the proportions of parous females of either species were not significantly different between the two villages (*p* > 0.05) but was slightly higher in *An. funestus* (0.50) than in *An. arabiensis* (0.42). Analysis of the 2020 data determined that *An. funestus* contributed 97.7% of all malaria transmitted in households in these two villages.

**Conclusions:**

In contexts where individual vector species mediate most of the pathogen transmission, it may be most appropriate to pursue a species-focused approach to better understand the ecology of the dominant vectors and target them with effective interventions to suppress transmission. Despite the ongoing efforts on tackling malaria in the two study villages, there is still persistently high *Plasmodium* infection prevalence in local populations of *An. funestus*, which now carry ~97% of all malaria infections and mediates intense year-round transmission. Further reduction in malaria burden in these or other similar settings requires effective targeting of *An. funestus.*

## 1 Introduction

Starting two decades ago when more than a million lives were being lost to malaria yearly (World Health Organization, 2000), there have been significant gains, including ~60% reduction in deaths, despite doubling of Africa's population (World Health Organization, 2019). Insecticide-treated nets (ITNs), indoor residual spraying (IRS) and effective case management have contributed greatly to these gains (Bhatt et al., 2015; Steketee and CCC., 2010). The persisting malaria transmission in most endemic settings is now thought to be largely due to widespread insecticide resistance among major malaria vectors (Matowo et al., 2017; Kaindoa et al., 2017; Casimiro et al., 2006; Cuamba et al., 2010), behavioral adaptation of malaria vectors to indoor interventions (Russell et al., 2011; Sougoufara et al., 2014; Moiroux et al., 2012), parasite resistance to anti-malarial drugs (Ashley et al., 2014; Dondorp et al., 2010), certain human behaviors and activities (Matowo et al., 2013; Finda et al., 2019; Monroe et al., 2020) and asymptomatic infections in older age groups such as school-aged children (Andolina et al., 2021). Other factors such as inadequate environmental sanitation, poor housing conditions and low household incomes may also be perpetuating risk in many endemic villages (Kaindoa et al., 2018; Liu et al., 2014), despite more than two decades of widespread ITN use.

In rural south-eastern Tanzanian villages, where ITNs have been widely used for >20 years, malaria transmission intensities have greatly reduced, in some cases from highs of >300 infectious bites per person per year (ib/p/y) in early 2000s to new averages below 30 ib./p/y (Okumu and Finda, 2021). These gains have been mostly attributed to widespread use of ITNs and effective case management (Okumu and Finda, 2021; Finda et al., 2018). Today, malaria transmission remains highly varied over small distances (Okumu and Finda, 2021; Russell et al., 2010), and recent parasite surveys have demonstrated ranges of <1% prevalence in urban centers of Ifakara town (<150 m above sea level) to highs of >40% in some higher altitude villages (~300 m above sea level) just 30 km away (Swai et al., unpublished).

Recent studies in Ulanga and Kilombero districts, have demonstrated that *Anopheles funestus* alone now contributes nearly nine in every ten new infections, even in villages where it is outnumbered by *Anopheles arabiensis* (Kaindoa et al., 2017; Lwetoijera et al., 2014). The species is also strongly resistant to pyrethroids used in the ITNs (Pinda et al., 2020), survives much longer than its counterparts (Kaindoa et al., 2017; Ngowo et al., 2021) and is strongly adapted to primarily bite humans over other vertebrates (Takken and Verhulst, 2013). Historically, *Anopheles gambiae* sensu stricto dominated transmission in Ulanga and Kilombero districts, with the *An. funestus* being another important vector during the dry season (Charlwood et al., 2000). However, *An. gambiae* s.s population and contribution to the malaria transmission in these districts started to decline following introduction of the ITNs (Russell et al., 2010; Lwetoijera et al., 2014), and reached undetectable level by 2012 (Lwetoijera et al., 2014). Recent studies have confirmed absence of the *An. gambiae* s.s in these districts, leaving the sibling species *An. arabiensis* at large (Kaindoa et al., 2017; Kaindoa et al., 2018; Ngowo et al., 2017; Limwagu et al., 2019). While no detailed analysis has been conducted, it is hypothesized that villages currently having the greatest malaria prevalence are those with highest densities of *An. funestus*. Moreover, given its preference for permanent and semi-permanent aquatic habitats which last far longer than the rainy season (Nambunga et al., 2020), this species is thought to be important for year-long transmission of malaria.

This current analysis was conducted in two villages, previously identified as having high densities of *An. funestus* (Kaindoa et al., 2019), and coincidentally having very high malaria prevalence estimates (Swai et al., unpublished). The aim was to analyze and compare seasonal prevalence of *Plasmodium* sporozoite infections in the main malaria vectors, *An. funestus* and *An. arabiensis* following nearly two decades of widespread ITN use.

## 2 Methods

### 2.1 Study area

**Fig. 1:**
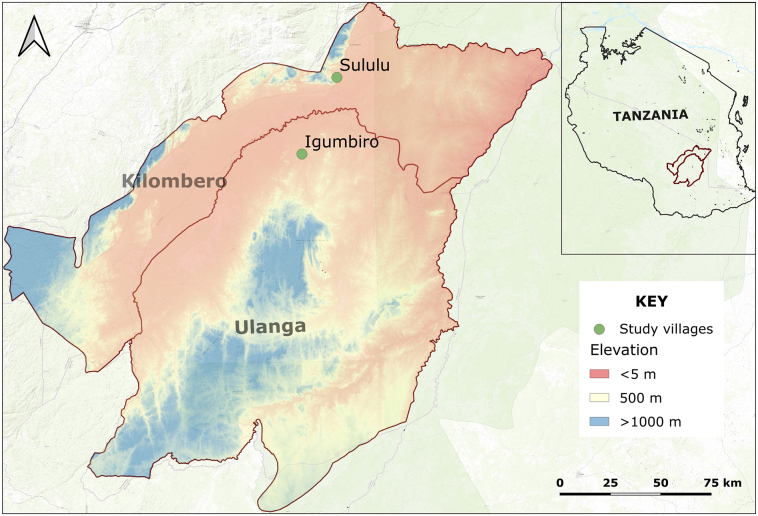
Map showing villages in the Kilombero valley, south-eastern Tanzania where adult *Anopheles funestus* and *Anopheles arabiensis* mosquitoes were collected.

The study was done in Ulanga and Kilombero districts, in the Kilombero valley in south-eastern Tanzania (Fig. 1). Mosquitoes were collected from two villages namely Sululu in Kilombero district (8.00324°S, 36.83118°E) and Igumbiro in Ulanga district (8.35021°S, 36.67299°E). Average annual rainfall was 1200–1600 mm and mean annual temperatures were 20–32 °C (Ngowo et al., 2017). Most residents here are subsistence farmers, cultivating mainly rice but also other crops such as maize, beans and sweet potatoes (Swai et al., 2016). According to 2012 national population census, Igumbiro and Sululu villages are within wards with the population of 16,329 and 9048 respectively (National Bureau of Statistics, 2013). Sululu village sits on higher altitude above sea level compared to Igumbiro village (Fig. 1).

### 2.2 Mosquito collection and processing

Mosquitoes were routinely collected from January 2018 to December 2020. In each village, sampling was done in 10–20 houses for five nights each week from January 2018 to January 2020, as part of the ongoing project that focused mainly on *An. funestus* mosquitoes. However, from March 2020 only three households were sampled nightly for four nights each week to December 2020, as part of another project focused on both *An. funestus* and *An. arabiensis* mosquitoes. Herein, data from both aforementioned projects were combined and analysed. All collections were done indoors, CDC light traps (Mboera et al., 1998) and Prokopack aspirators (Maia et al., 2011) were used to sample host-seeking and resting mosquitoes respectively. Female mosquitoes were identified and sorted by taxa and physiological state (Gillies and De Meillon, 1968), after which *An. arabiensis and An. funestus* were packed in batches of ten mosquitoes in 1.5 ml microcentrifuge tubes for circumsporozoite enzyme-linked immunosorbent assays (ELISA) (Beier et al., 1990), of which only a pool of heads and thoraces were used. Equal numbers of *An. arabiensis* and *An. funestus* mosquitoes collected from March 2020 to December 2020 (i.e. recent project) were subjected to ELISA, whilst the additional ELISA results were retrieved from the previous project (i.e. January 2018–January 2020) that only screened *An. funestus*. The ELISA lysates were boiled for 10 min at 100 °C to eliminate heat-labile non *Plasmodium* protozoan antigens that will render false positivity (Durnez et al., 2011). A sub-sample of the mosquitoes collected from March 2020 to December 2020 were subjected to dissection to assess parity status as described by Kaindoa et al (Kaindoa et al., 2017). Recent studies in the area had determined that indoor collections of *An. gambiae* s.l. consisted only of *An. arabiensis* (100%), and those of *An. funestus* group were mainly *An. funestus* s.s (Kaindoa et al., 2017; Ngowo et al., 2017).

### 2.3 Statistical analysis

R statistical software version 3.6.0 (R Development Core Team R, 2019) was used to execute a simple generalized linear model (GLM) with binomial distributions to examine odds of sporozoite-infected *Anopheles* and also parous mosquitoes. Generalized linear mixed models (GLMM) were used to model the abundance of different mosquito species. Here the number of mosquitoes caught were modelled as a response variable using a negative binomial variate to account for the over-dispersion in the number of catches. Sampling location and season were included as the fixed terms. To account for the unexplained variation between sampling days, sampling dates nested within a month of the collection was included as random term. Relative risk and their corresponding 95% CI were reported. Entomological inoculation rate (EIR) was estimated as a function of a biting rate and a proportion of mosquitoes tested positive with *Plasmodium* sporozoite. The annual EIR is estimated by multiplying a daily EIR with 365 (Annual EIR = daily biting rate ✕ sporozoite rate ✕ 365). The adjusted annual EIR was estimated by the same function except that, the corrected biting rate was used in the place of daily biting rate as described by Kaindoa et al (Kaindoa et al., 2017). Herein, the dry season comprises of months from January to May, and from June to December for the rainy season.

## 3 Results

A total of 306,589 mosquitoes were collected with 53.8% (*n* = 165,058) being *An. funestus* mosquitoes. A sub-sample of the collected mosquitoes (*n* = 10,241) were analysed for *Plasmodium* sporozoite infections, of which 23.3% (*n* = 2382) were *An. arabiensis* and 76.7% (*n* = 7859), *An. funestus*. Of 7859 *An. funes*tus tested, 4.6% (*n* = 365) were positive for *Plasmodium* sporozoites in the salivary glands. On the contrary, only 0.4% (*n* = 9) of the 2382 *An. arabiensis* tested were positive. Though the sporozoite prevalence during rainy season appeared slightly higher for both vectors (Table 1), this analysis showed no statistically significant difference in the sporozoite prevalence for *An. funestus* between villages or seasons (*p* > 0.05). Both *An. arabiensis* and *An. funestus* mosquitoes had higher sporozoite prevalence in Sululu than in Igumbiro village (Table 1).

**Table 1:** Results of the multivariate analysis of *Plasmodium falciparum* sporozoites infectivity in *Anopheles funestus* and *Anopheles arabiensis* mosquitoes by village and season.

Species	Village season		Total (N)	Sporozoite prevalence n (%)	OR (95% LC-UC)	*p*-value
*Anopheles funestus*	Igumbiro	Dry	2568	114 (4.3)	1	0.89
		Rainy	1346	61 (4.5)	1.02 (0.74–1.40)	
	Sululu	Dry	1857	85 (4.6)	1	0.51
		Rainy	2088	105 (5.0)	1.10 (0.82–1.45)	
*Anopheles arabiensis*	Igumbiro	Dry	1724	3 (0.2)	1	0.19
		Rainy	127	1 (0.8)	4.55 (0.47–44.09)	
	Sululu	Dry	448	5 (1.1)	1	0.33
		Rainy	83	0 (0.0)	0.99 (0.97–1.01)	

*Percentage sporozoite-prevalence = Sporozoite positive (n)/Total number of mosquitoes analysed (N).

The parity dissections determined that 50.3% of the *An. funestus* (n/*N* = 80/159) and 41.8% of the *An. arabiensis females* (n/*N* = 66/158) were parous (Table 2). There was no statistical difference in parity between villages for either species (Table 2). Proportion of *An. funestus* positive for *Pf* sporozoite was high in both seasons (Table 1), and both villages recorded high densities of both vector species throughout the year (Table 3). Analysis of the year-to-year data showed that in 2020, 97.7% of all malaria transmission events were mediated by *An. funestus*, and that *An. arabiensis* played only a minor role (Table 4). (See Table 5.)

**Table 2:** Results of the multivariate analysis of parity in *Anopheles funestus* and *Anopheles arabiensis* mosquitoes by village.

Species	Village	Total (N)	Parous n (%)	OR (95% LC-UC)	p-value
*Anopheles funestus*	Igumbiro	79	39 (49.4)	1	0.81
	Sululu	80	41 (51.3)	1.08 (0.58–2.01)	
*Anopheles arabiensis*	Igumbiro	78	35 (44.9)	1	0.44
	Sululu	80	31 (38.8)	0.78 (0.41–1.46)	

*Percentage parous = parous (n)/Total number of mosquitoes examined (N).

**Table 3:** Results of the multivariate analysis of biting densities of *Anopheles funestus* and *Anopheles arabiensis* mosquitoes by village and season.

Species	Village	Season	Total	Mean ± 2SE	RR (95% LC-UC)	p-value
*Anopheles funestus*	Igumbiro	Dry	35,496	11.4 ± 0.5	1	0.51
		Rainy	20,121	11.2 ± 0.4	1.16 (0.74–1.81)	
	Sululu	Dry	47,979	10.1 ± 0.4	1	0.08
		Rainy	53,603	20.1 ± 1.0	1.68 (0.94–3.0)	
*Anopheles arabiensis*	Igumbiro	Dry	57,336	18.5 ± 0.8	1	0.70
		Rainy	30,813	17.2 ± 0.6	1.12 (0.63–1.98)	
	Sululu	Dry	17,483	3.7 ± 0.3	1	<0.001
		Rainy	33,517	12.6 ± 0.6	3.83 (2.35–6.24)

**Table 4:** Infectious status of *Anopheles funestus* and *Anopheles arabiensis* mosquitoes collected from 2018 to 2020.

	*Anopheles funestus*			*Anopheles arabiensis*
	2018	2019	2020	2020
Total number of mosquitoes collected by CDC light trap	73,237	72,661	9795	12,772
Total number of trap nights	6206	4287	476	476
Biting rate per night	11.8	16.95	20.58	3.08
Relative efficiency (CDC-LT) relative to HLC (derived from Okumu et al (Okumu et al., 2008))	0.68	0.68	0.68	0.3
Corrected biting rate	17.35	24.93	30.26	10.27
Total number of mosquitoes analysed for *Plasmodium falciparum* circumsporozoite protein (CSP) - (S)	3641	1604	1228	1466
Total number of sporozoite positive mosquitoes (s)	178	64	77	6
Sporozoite prevalence (s/S)	0.05	0.04	0.06	0.0041
**Annual EIR (adjusted)**	**316.63**	**363.98**	**662.69**	**15.37**
**% EIR contribution for the year 2020 (adjusted)**			**97.7%**	**2.3%**

*Annual EIR (adjusted) = Corrected biting rate ✕ Sporozoite rate ✕ 365.

*The data presented here excluded samples collected by Prokopack aspirators.

**Table 5:** *Plasmodium* sporozoite infectivity by village, mosquito species and month.

Month	Sululu		Igumbiro	
	*An. funestus*	*An. arabiensis*	*An. funestus*	*An. arabiensis*
Jan-18%(N)	–	–	0% (1)	–
Feb-18%(N)	–	–	0% (15)	–
Mar-18%(N)	15.1% (126)	–	6.7% (150)	–
Apr-18%(N)	11.1% (144)	–	5.7% (159)	–
May-18%(N)	3.5% (666)	–	1.6% (569)	–
Jun-18%(N)	5.8% (365)	–	5.2% (250)	–
Jul-18%(N)	1.6% (131)	–	0% (16)	–
Aug-18%(N)	5.4% (184)	–	4.2% (120)	–
Sep-18%(N)	7% (172)	–	4.2% (262)	–
Oct-18%(N)	22.2% (9)	–	13% (23)	–
Nov-18%(N)	0% (40)	–	3.7% (82)	–
Dec-18%(N)	5.7% (88)	–	6.8% (44)	–
Jan-19%(N)	6% (332)	–	0% (22)	–
Feb-19%(N)	2.3% (129)	–	7.1% (85)	–
Mar-19%(N)	3.9% (127)	–	6.7% (15)	–
Apr-19%(N)	4% (125)	–	33.3% (6)	–
May-19%(N)	2.6% (389)	–	4.7% (106)	–
Jun-19%(N)	2.2% (46)	–	10% (10)	–
Jul-19%(N)	0% (2)	–	–	–
Aug-19%(N)	0% (1)	–	0% (50)	–
Sep-19%(N)	0% (3)	–	0% (19)	–
Oct-19%(N)	4.9% (61)	–	0% (23)	–
Nov-19%(N)	7.7% (26)	–	–	–
Dec-19%(N)	4.8% (42)	–	0% (10)	–
Jan-20%(N)	–	–	0% (1)	–
Mar-20%(N)	8% (50)	0% (83)	8.8% (217)	0.8% (127)
Jun-20%(N)	8.8% (170)	0% (11)	8.2% (403)	0.5% (397)
Jul-20%(N)	0% (126)	0% (107)	4.5% (313)	0% (315)
Aug-20%(N)	6.6% (76)	1.7% (59)	0% (140)	0% (280)
Sep-20%(N)	0% (204)	0.9% (106)	0% (413)	0% (281)
Oct-20%(N)	5.9% (85)	4.2% (72)	5.9% (153)	0% (276)
Nov-20%(N)	0% (25)	0% (88)	8% (237)	0.6% (174)

## 4 Discussion

Recent evidence has shown that *An. funestus* now mediates most of the ongoing malaria transmission in many countries in East and Southern Africa (Kaindoa et al., 2017; Burke et al., 2019; McCann et al., 2014). In rural south-eastern Tanzania, this species carries >85% of all malaria infections even in villages where it occurs at lower frequencies than *An. arabiensis* (Kaindoa et al., 2017; Finda et al., 2018; Swai et al., 2019). Malaria transmission has declined significantly in the Kilombero valley since 2000, mostly due to the wide coverage of ITNs and effective case management (Okumu and Finda, 2021; Russell et al., 2010).

This short report presents an analysis of mosquitoes collected between 2018 and 2020 in two villages previously identified as having very high densities *of An. funestus*. It had been hypothesized that the proven dominance of *An. funestus* in the valley, coupled with the high densities of this vector species, as well as its strong pyrethroid-resistance status and greater survival in nature may lead to high residual transmission burden in the relevant villages, and that this situation may make malaria transmission control much more difficult than elsewhere. This analysis confirms the high parity rates but further presents two surprising findings. First is the extremely high *Plasmodium* infection prevalence in the *An. funestus* mosquitoes over the three years of sampling, averaging 4.6% despite high ITN use in the areas. Given the nightly mosquito catches by CDC light traps, this translates to 447.8 infectious bites/person/year, which is considerably higher than most recent estimates (Kaindoa et al., 2017; Finda et al., 2018). The second finding was that the high infection prevalence rates were consistent throughout dry and rainy season for the entire duration of the study. This suggests that *An. funestus* not only plays an important role in transmission but also that it mediates transmission throughout the whole year. There were months with very high transmission and months with very low transmission, but when data was aggregated by season, there was no significant difference in transmission intensities. Additional studies will be necessary to understand the dry season ecology *of An. funestus* and the factors driving transmission at different times of the year, so as to design effective tools towards reducing malaria transmission. Additionally, the higher sporozoite prevalence observed in *An. arabiensis* in one of the villages (i.e. Sululu) corroborate recent finding (Swai et al., unpublished) that indicate geographical heterogeneity in transmission over small distances. Thus, understanding local vectors' ecology is crucial for control measures. Polymerase chain reaction (PCR) may have greater sensitivity in detection of *Plasmodium* sporozoite, however, these improvements are marginal and practically irrelevant in areas with moderate to high transmission such as south-eastern Tanzanian districts of Ulanga and Kilombero.

Kaindoa et al. (Kaindoa et al., 2017) reported *An. funestus* mosquitoes carrying 86% of malaria infections in Kilombero valley. Lwetoijera et al. also demonstrated significant role of *An. funestus* following the decline of *An. gambiae,* formerly the most important vector in the valley (Lwetoijera et al., 2014). Since then, several other studies have confirmed the dominance of *An. funestus* in this area (Okumu and Finda, 2021). In addition, reports of higher entomological inoculation rates by *An. funestus* have also been documented elsewhere (Cohuet et al., 2004; Trape et al., 2014; Antonio-Nkondjio et al., 2002), however, the rates were not as high. Other studies have also shown the strong pyrethroid resistance, and that while its aquatic ecology is still poorly understood, it occupies perennial habitats which remain water-filled most of the year (Nambunga et al., 2020). This ecological characteristic may explain its dominance in densities and transmission activity throughout the year. It is clear therefore that efforts to further reduce malaria transmission by this vector must consider specific measures targeting *An. funestus* so as to strongly diminish its potential.

Opportunities for improved control of *An. funestus* in the area may include new generation ITNs with synergists or multiple actives (Gleave et al., 2018; Protopopoff et al., 2018; Pennetier et al., 2013; Corbel et al., 2010), or use of larval source management, which would be effective even against the pyrethroid-resistant populations. Fortunately, the current National Malaria Strategic Plan (NMSP) of Tanzania encourage implementation of larviciding in rural settings (Ministry of Health and Social Welfare, 2022). Coupled with recent findings of Nambunga et al. (Nambunga et al., 2020) which indicate that aquatic habitats of *An. funestus* mosquitoes in Kilombero valley falls within few, fixed and findable criterion of World Health Organization (World Health Organization, 2013). Thus, provide possibilities of a cost-effective and plausible species-specific intervention to significantly reduce malaria transmission in this valley. Additionally, susceptibility of *An. funestus* towards organophosphate notably pirimiphos methyl poses a potential opportunity for control. Pirimiphos methyl is already approved and used for IRS in Tanzania (Mashauri et al., 2017; Haji et al., 2015), thus there is a need to design cost-effective tools that may exploit the possibility of using pirimiphos methyl.

## 5 Conclusion

This study demonstrates that despite the widespread use and overall impact of ITNs, there is still persistently high *Plasmodium* infection prevalence in the dominant malaria vector, *An. funestus*, causing intense year-round malaria transmission in the study villages. Further reduction in malaria burden in this and similar settings thus requires effective targeting of *An. funestus*. The study also demonstrates that in certain contexts such as these, where one species is mediating most of the pathogen transmission, there could be significant potential in pursuing a species-specific approach for vector control by investigating and targeting the dominant vector species to suppress transmission.

## Ethical approval and consent to participate

Ethical approvals for this project were obtained from Ifakara Health Institute's Institutional Review Board (Ref. IHI/IRB/No: 007–2018) and the Medical Research Coordinating Committee (MRCC) at the National Institute for Medical Research, in Tanzania (Ref: NIMR/HQ/R.8a/Vol. IX/2895). Written consents were sought from all participants of this study, after they had understood the purpose and procedure of the discussions.

## Consent for publication

Permission to publish this study was obtained from National institute for Medical Research, in Tanzania (NIMR/HQ/P.12 VOL XXXII/144).

## Data availability

Data used to generate our findings can be accessed upon reasonable request to the corresponding author.

## Funding statement

This work was supported by the 10.13039/100010269Wellcome Trust International Masters Fellowship in Tropical Medicine & Hygiene (Grant No. 212633/Z/18/Z) awarded to SAM. This work was also supported by 10.13039/100000865Bill and Melinda Gates Foundation (Grant Number: OPP1177156) and 10.13039/100000011Howard Hughes Medical institute (Grant number: OPP1099295) both awarded to FOO.

## Authors contributions

SAM, FOO, HSN and EEH were involved in study design. SAM, EEH, HSN, JK, HB, KK and MK were involved in data collection. SAM and HSN conducted data analysis. SAM wrote the manuscript. FOO, HSN, EWK and EEH provided thorough review of the manuscript. All authors read and approved the final manuscript.

## Declaration of Competing Interest

Authors declare no conflict of interest.
